# Feasibility and Safety of Combined Robot-Assisted Gait Training and Transcutaneous Spinal Cord Stimulation in Pediatric Incomplete Spinal Cord Injury: A Case Series

**DOI:** 10.3390/children13070859

**Published:** 2026-06-27

**Authors:** Javier Merino-Andrés, Ana Onate-Figuerez, Soraya Pérez-Nombela, Julio Gómez-Soriano, Elisa López-Dolado, Olivia Martín-Nieto-Ríos, Inés García de la Torre-Soto, Diego Serrano-Muñoz

**Affiliations:** 1 Toledo Physiotherapy Research Group (GIFTO), Faculty of Physiotherapy and Nursing, Universidad de Castilla-La Mancha, 45071 Toledo, Spain; javier.merino@uclm.es (J.M.-A.); ana.onate@uclm.es (A.O.-F.); julio.soriano@uclm.es (J.G.-S.); diego.serrano@uclm.es (D.S.-M.); 2 Toledo Physiotherapy Research Group (GIFTO), Instituto de Investigación Sanitaria de Castilla-La Mancha (IDISCAM), 45004 Toledo, Spain; 3 Pediatric Rehabilitation Department, Hospital Nacional de Parapléjicos (SESCAM), 45004 Toledo, Spain

**Keywords:** pediatric spinal cord injury, child, transcutaneous spinal cord stimulation, robot-assisted gait training, gait

## Abstract

**Highlights:**

**What are the main findings?**

- The combined robot-assisted gait training and transcutaneous spinal cord stimulation intervention was feasible and safe in children with incomplete spinal cord injury, with high adherence and only mild, mostly transient adverse events.
- All participants improved gait speed, with clinically meaningful changes in most cases, while secondary outcomes showed heterogeneous effects as preliminary data.

**What are the implication of the main findings?**

- Combined neuromodulation and robotic gait training may represent a promising rehabilitation strategy to enhance locomotor recovery in pediatric spinal cord injury.
- These findings support the need for larger controlled trials to confirm efficacy and optimize treatment protocols.

**Abstract:**

**Background/Objectives:** Pediatric spinal cord injury (SCI) is a rare yet highly disabling condition associated with substantial functional and psychosocial impairments. Although robot-assisted gait training (RAGT) and transcutaneous spinal cord stimulation (tSCS) have independently demonstrated promising results, evidence regarding their combined use in pediatric SCI is limited. This study aimed primarily to assess the feasibility and safety of combining RAGT with tSCS in children with incomplete SCI, and secondarily to explore its effects on clinical outcomes. **Methods:** A case series study was conducted that included three pediatric participants (<18 years) with chronic incomplete SCI (AIS C–D). Participants completed five consecutive sessions of RAGT using a Lokomat device combined with tSCS applied at the lumbosacral level. Each session consisted of 30 min of gait training, including 20 min of concurrent electrical stimulation. Safety was assessed through adverse-event monitoring and pain evaluation. Clinical outcomes included gait speed (10MWT), trunk control (SATCo), lower-limb strength (LEMS), and spasticity (MAS). **Results:** Session adherence reached 100%. Skin erythema was the most frequently reported adverse event and showed a clear association with tSCS. All participants demonstrated improvements in gait speed, with two exceeding the minimal clinically important difference. Secondary results showed similar outcomes for spasticity, strength, and trunk control, with no clinically meaningful changes in any case. **Conclusions:** The combined application of RAGT and tSCS appears to be a feasible and safe intervention for children with incomplete SCI. Preliminary findings suggest potential benefits in gait speed, thereby supporting the need for further investigation with larger samples and controlled study designs.

## 1. Introduction

Pediatric spinal cord injury (SCI), generally defined as injury to the spinal cord occurring before 18 years of age, lacks a universally accepted age cutoff [1]. Although the International Spinal Cord Society (ISCoS) has proposed age limits, these have not been formally established in the literature. Despite its relatively low incidence, pediatric SCI represents a devastating condition associated with high morbidity, long-term dependence, and profound physical, psychological, and social consequences for affected children and their families [2,3,4,5].

Epidemiological data vary across regions, but pediatric SCI remains a rare condition of both traumatic and non-traumatic origin, representing a small proportion of total SCI cases [1,3].

Neurological level and injury severity, commonly classified using the American Spinal Injury Association (ASIA) Impairment Scale (AIS), are determinants of functional recovery [6,7]. This is particularly relevant in pediatric populations, where incomplete injuries represent the most common presentation, as the preservation of residual neural pathways confers greater potential for recovery [8,9,10,11]. In addition, the developing nervous system may offer enhanced neuroplasticity, supporting rehabilitation-induced functional gains [12].

Restoring independent mobility is a primary goal of rehabilitation, given its critical role in physical development and participation for children with SCI [13,14]. This priority is also reflected in patient-reported outcomes, with parents and caregivers identifying walking and mobility as major concerns, while adolescents emphasize lower-limb function as well as bladder and bowel control [15]. However, pediatric SCI rehabilitation remains challenging due to variability in access to specialized care and the lack of standardized treatment approaches [16,17].

Current strategies emphasize high-intensity, task-specific training to promote activity-dependent neuroplasticity [18,19]. In this context, robot-assisted gait training (RAGT) has been increasingly used, although functional outcomes remain inconsistent, suggesting that additional therapeutic approaches may be required [20].

Transcutaneous spinal cord stimulation (tSCS) is a noninvasive neuromodulation technique that delivers electrical currents via surface electrodes to modulate spinal network excitability [21]. It has gained increasing attention in recent years and has shown promising effects when combined with gait training interventions [22]. tSCS enhances spinal circuit excitability primarily through activation of dorsal root afferents, thereby modulating motor thresholds and facilitating voluntary movement [23].

Spinal cord stimulation has shown promising results in adult populations by modulating neural networks involved in motor control and improving gait recovery [22]. In pediatric populations, however, evidence remains limited, despite the potentially greater responsiveness associated with the enhanced neuroplasticity of the developing nervous system. Most SCI rehabilitation research has been conducted in adults, and high-quality studies identifying optimal neurotherapeutic interventions for children are still lacking. Nevertheless, the translation of spinal cord stimulation to pediatric populations has begun, with pilot studies indicating that these approaches are safe and feasible, thereby providing essential preliminary evidence to support further investigation [24,25,26,27].

Emerging evidence suggests that tSCS may improve motor and functional outcomes in children with neurological disorders. In pediatric SCI, reported benefits include improvements in trunk control, posture, and functional mobility, with small case series indicating gains in walking performance without significant adverse effects [23,25,28].

Preliminary findings in other pediatric populations, such as cerebral palsy, further support the potential of tSCS to enhance motor performance, although current evidence remains limited and heterogeneous. To date, there is a lack of robust evidence regarding the efficacy of combining tSCS with rehabilitation training in pediatric SCI.

Accordingly, the primary objective of this study was to evaluate the feasibility and safety of an intervention combining robot-assisted gait training with body-weight support and tSCS in children with incomplete SCI. The secondary objective was to examine the effects of this combined intervention on relevant clinical outcomes.

## 2. Materials and Methods

A case series study was conducted and approved by the Clinical Research Ethics Committee of the Complejo Hospitalario de Toledo (approval number 1147), with informed consent obtained from the parents or legal guardians of all participants.

Participants with pediatric SCI were recruited from the Pediatric Rehabilitation Service of the National Hospital for Paraplegics in Toledo. Subjects met the following predefined inclusion criteria: age < 18 years; thigh length of 21–35 cm (greater trochanter to knee joint line); lower-leg length of 17–28 cm (knee joint line to floor while wearing shoes); chronic motor incomplete SCI (AIS C–D); neurological level of injury between C1 and T11; ability to understand and follow instructions; and written informed consent signed by parents, primary caregivers, or legal representatives.

Exclusion criteria included intolerance to electrical stimulation; Cobb angle > 60°; moderate to severe hypertonia (Modified Ashworth Scale (MAS) ≥ 2); peripheral neurological injury affecting the lower limbs; vertebral arthrodesis or metallic implants at vertebral levels T11–T12; musculoskeletal or joint injury of the lower limbs; presence of any implanted electronic device (e.g., pacemaker, baclofen pump); skin alterations or pressure injuries in areas of contact with the Lokomat harness straps; irreducible hip or knee flexion contractures or lower-limb arthrodesis; osteoporotic fractures within the previous 2 years; history of lower-limb surgery within the previous 6 months; concomitant medical conditions (cardiac, respiratory, renal, hepatic, oncological, or similar); and uncontrolled epilepsy.

Throughout the intervention protocol, patients completed a total of five consecutive sessions consisting of RAGT using the Lokomat combined with tSCS. Each session consisted of Lokomat gait training for 30 min or until a distance of 1 km was completed, whichever was achieved first for each participant. Training parameters, including body-weight support, treadmill speed, guidance force, and therapist assistance, were individually adjusted according to each participant’s clinical status, tolerance, and performance. During the central 20 min of each session, tSCS was applied at the lumbosacral level over five consecutive days. tSCS was delivered using a Myomed 632 device (Enraf-Nonius, The Netherlands), which generated a symmetrical rectangular biphasic current at a frequency of 30 Hz and a pulse width of 1 ms (phase duration 0.5 ms, interpulse interval 0 ms). Three self-adhesive pre-gelled flexible carbon surface electrodes (5 × 5 cm, ValuTrode, Axelgaard Manufacturing Co., Fallbrook, CA, USA) were used.

**Figure 1:**
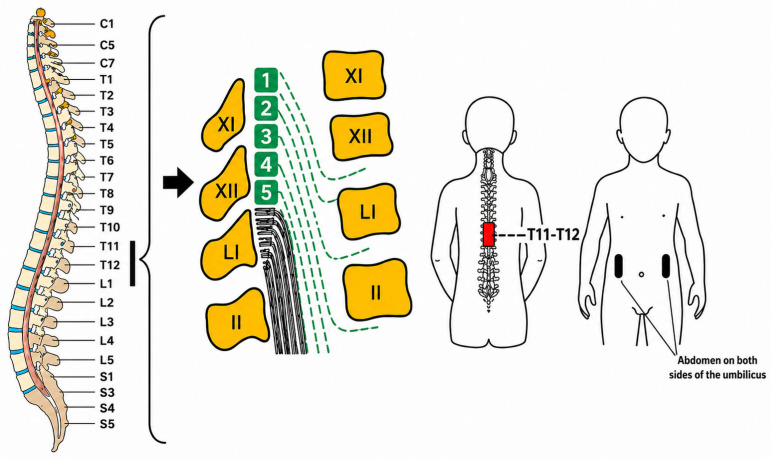
Electrode arrangement.

The anode was positioned over the lumbosacral spinal cord region along the midline between the T11–T12 spinous processes, while two interconnected cathodes were placed symmetrically on the abdomen on both sides of the umbilicus. This montage was selected to optimize current distribution over the spinal segments and has been previously described in the literature [22]. See the electrode arrangement in Figure 1.

Stimulation intensity was individually adjusted in each session and increased gradually up to the participant’s sensory tolerance threshold, without exceeding comfort limits, in order to ensure safety and tolerability in the pediatric population. In addition, each subject underwent conventional treatment according to their individual goals.

To assess the safety and feasibility of the combined interventions, adverse events and pain were recorded. Monitoring of both adverse responses and pain was continuous throughout the sessions. Adverse events were assessed using an ad hoc questionnaire developed for this study and administered 24 h after each session. Pain was assessed using the Wong–Baker FACES Pain Rating Scale, which is familiar to and is frequently used by healthcare professionals. It consists of six hand-drawn facial expressions arranged horizontally, illustrating a spectrum of pain intensity. On the left, a smiling face represents 0 = no pain, while on the right, a crying face represents 10 = the worst possible pain. The original scoring method, which ranged from 0 to 5, was later adapted to a 0–10 scale to make it more intuitive and easier to use [29].

To assess the effects on gait speed, the 10-meter walk test (10MWT) was administered before and after the intervention; this measure has demonstrated reliability and validity in individuals with SCI. It assesses walking speed by measuring the time required to walk 10 m, allowing for physical assistance, orthoses, or any necessary assistive devices. The 10MWT is performed using a dynamic start, providing a 2 m acceleration phase before the timed 10 m section, followed by a 2 m deceleration phase after completing the measured distance [30]. Trunk control—static, dynamic, and reactive—was evaluated using the Segmental Assessment of Trunk Control (SATCo), a tool developed for pediatric populations that evaluates trunk control in sitting incrementally across seven levels of support (from shoulder to no support). It also evaluates three types of control and is sensitive and responsive to therapeutic interventions in the pediatric SCI population [31]. Lower-extremity strength was assessed using the lower-extremity motor score (LEMS). Lower-limb strength was evaluated in children by examining specific muscle groups, including the hip flexors, knee extensors, ankle dorsiflexors, toe extensors, and ankle plantar flexors. Muscle strength was graded on a scale from 0 to 5 based on the results of the manual muscle test, and the sum of the individual muscle scores for the lower extremities was used as the overall final score [19]. Finally, lower-limb hypertonia was evaluated using the MAS. It is a numerical scale that grades spasticity from 0 to 4, with 0 being no resistance and 4 being a limb rigid in flexion or extension [30]. All tests were measured pre-intervention and immediately after the intervention ended.

Given the case series design, changes were primarily reported at the individual level using descriptive statistics. Outcomes were compared against the minimal clinically important difference (MCID) when available. For the 10MWT, the MCID was set at 0.06 m/s [32,33]. For the MAS, the MCID was set at 0.45 ± 0.73 [34]. For the SATCo, a change of 2 points was considered clinically significant following the intervention [31].

## 3. Results

Three participants were included in the present study. Demographic characteristics are summarized in Table 1. Session compliance and attendance reached 100%, with 15 out of 15 planned sessions completed. All included participants met the eligibility criteria, and none presented scoliosis exceeding the exclusion threshold (Cobb angle > 60°).

The results related to adverse events, including their distribution, severity grading, and relationship to tSCS, are presented in Table 2. A total of 18 adverse events were recorded across the 15 intervention sessions. Skin erythema was the most frequently reported adverse event, accounting for 53% of all events, and showed a definitive relationship with tSCS in 87.5% of cases.

**Figure 2:**
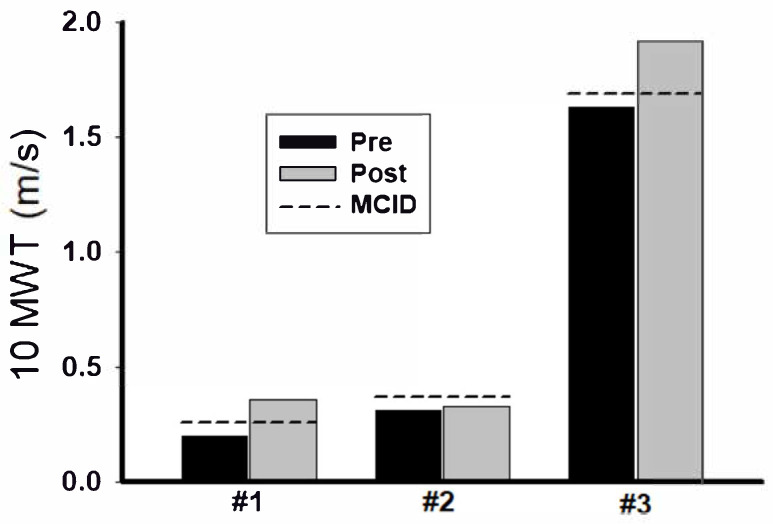
Results obtained for the 10-meter walk test (10MWT).

Pain was reported in only one session by a single participant, who rated the perceived pain as 6 during the first stimulation and training session. Regarding gait outcomes, improvements in the 10MWT were observed in all three participants; two of them exceeded the MCID. The results for the 10MWT are presented in Figure 2 and in Table 3.

With respect to the remaining clinical variables, findings showed a reduction in lower-limb hypertonia in one participant, obtaining the MCID for this outcome. The LEMS improved in one participant, worsened in another, and remained unchanged in the third. Finally, no changes were observed in the SATCo scores for any participant across any of its subdimensions or in the MAS. The results are presented in Table 3.

In addition, qualitative observations reported by family members or primary caregivers were collected for each participant. Case 1: “*Shows better trunk control*”. Case 2: “*Is more upright*”. “*Fell asleep earlier*”. “*Is more active and walked longer distances independently*”. “*Appears to have better bladder sphincter control*”. Case 3: “*Walks more lightly*”. “*Has urinated more frequently*”. “*Presented with abdominal skin redness*”.

## 4. Discussion

The primary objective of this study was to evaluate the feasibility and safety of combining RAGT with tSCS in a pediatric population with incomplete SCI. The most frequently observed stimulation-related adverse event was skin erythema at the electrode application site. Other adverse events included pain and/or a burning sensation during current stimulation that was directly related to electrical stimulation, but their frequency of occurrence was low. The remaining adverse events assessed were either not observed or were considered unlikely to be related to tSCS. Regarding the secondary objective of the study, all participants showed improvements in gait speed as measured by the 10MWT as preliminary data, with two of them achieving changes that were clinically meaningful.

To date, this is the first study to specifically analyze the safety of combining Lokomat-assisted gait training with tSCS in a pediatric SCI population. In the study by Comino-Suárez et al. [22], the combination of these two interventions was investigated in adults with SCI, showing that the most common adverse event was skin erythema at the tSCS electrode placement site. Similarly, with respect to the application of tSCS, three studies to date have assessed its safety in the pediatric SCI population. Keller et al. [25] reported findings comparable to those observed in the present case series, with participants describing a burning sensation at low stimulation intensities that resolved upon cessation of current stimulation. Skin erythema at the stimulation site was reported in 9 out of 22 evaluations (41%) and resolved by the following day.

Similarly, Singh et al. [28] demonstrated the safety of cervical tSCS application in a pediatric SCI population. Pain at the stimulation site was reported in three of seven participants; however, adjustment of stimulation intensity allowed participants to continue performing the prescribed functional activities. Optimal stimulation parameters for tSCS have not yet been established, so it is important to consider each subject’s perception of current intensity, requiring periodic readjustment of this parameter. Thus, during the current stimulation in this study, the current was gradually increased, and even when pain was perceived, the current intensity was reduced. Skin erythema was also observed in 19% of sessions, resolving within 24 h.

Neighbors et al. [23] evaluated the safety of tSCS for gait improvement in children with pediatric SCI following acute flaccid myelitis. Observed adverse effects were not attributable to stimulation itself but rather to pain induced by orthotic use during gait training. Only skin erythema related to electrode placement was reported following tSCS application.

With respect to functional outcomes, the present findings should be interpreted as exploratory. All participants showed improvements in gait speed (10MWT), with two achieving clinically meaningful changes. Comino-Suárez et al. also reported significant improvements in gait performance based on the 10MWT and the six-minute walk test outcomes [22]. Notably, such improvements were observed after completion of a 20-session stimulation program, whereas the present study employed a substantially lower number of sessions. Therefore, future controlled studies are needed to determine both the magnitude and durability of effects achieved through combined protocols with higher intervention dosages.

Ma et al. reported positive effects following a RAGT program on gait speed, cardiovascular function, energy expenditure, lower-limb strength, and center-of-pressure stability. Their protocol included 30 min Lokomat sessions combined with five weekly physiotherapy sessions of 30 min each over an 8-week period [19]. The smaller strength gains observed in the present study may be attributable to a lower RAGT dosage, despite Ma et al. also reporting only modest improvements in lower-limb strength [19].

In terms of MCID, the results of the present study align with existing literature. Neighbors et al. observed MCID in only one of four participants in their combined intervention study, while one additional participant showed improvement without reaching the MCID threshold, and two showed no improvement [23]. In contrast, Ma et al. reported substantial improvements surpassing the MCID in the experimental group receiving RAGT alone [19].

The combination of tSCS with RAGT, representing task-specific gait training, has been shown to be safe and effective in improving mobility-related outcomes in adults with SCI [22]. This combined intervention approach opens new therapeutic possibilities for the pediatric SCI population through the application of noninvasive neuromodulation. However, initiation of such interventions should be contingent on each participant’s ability to communicate adverse effects related to stimulation and to generate voluntary movement. Moreover, anatomical differences between adult and pediatric populations must be carefully considered to ensure optimal tSCS dosing and application parameters [24]. In this context, spatiotemporal neuromodulation may further enhance gait quality, weight-bearing capacity, and endurance [35].

Additionally, Keller et al. [25] observed improvements in postural control. These postural improvements contrast with the findings of the current study, likely due to the already adequate sitting motor control exhibited by the enrolled participants (ceiling effect at baseline). Furthermore, the duration of chronicity, along with previous rehabilitation interventions, may influence trunk control outcomes [31]. In the study by Singh et al. [28], participants also experienced improvements in hand strength and wrist extension after three days of stimulation.

The caregiver-reported changes in bladder function should be interpreted with caution and cannot be considered conclusive. However, they may be potentially related to the stimulation of lumbosacral spinal segments, which are involved in the neural control of lower urinary tract function.

Given the exploratory nature of this study and the absence of objective urological assessments, these interpretations remain speculative. Future studies should include standardized measures to better evaluate possible autonomic effects of tSCS.

Finally, the subjects’ perceptions during the intervention sessions were positive, as they expressed a sense of improvement in trunk motor control, perceived improvements during gait, and perceived improvements related to sphincter control.

### Limitations

The primary limitation of this study is the small sample size. For future studies, it would be advisable to increase the sample size, in addition to conducting clinical trials with a control group and a higher dosage protocol. As a case series, the results must be interpreted with caution; however, they represent an important preliminary step toward the design of larger clinical trials.

An additional limitation is the heterogeneity of the sample, particularly regarding age and clinical characteristics. Differences in neurodevelopmental stage may influence brain maturation and neuroplasticity following spinal cord injury. Furthermore, variability in injury level, severity, etiology, and time since injury may also have influenced the observed outcomes.

Previous research suggests that both developmental stage and injury characteristics can affect recovery trajectories and patterns of neural reorganization. Therefore, this heterogeneity may have contributed to variability in the results and should be considered when interpreting the findings. However, it may also enhance the ecological validity of the study, as it reflects the diversity of pediatric spinal cord injury populations.

A further limitation is the short follow-up period for each subject, as longer follow-ups are recommended to compare the intervention periods with the rest periods. Furthermore, a greater number of functional tests should be used to measure gait improvement. Another limitation of this study is that bladder-related outcomes were collected only through caregiver-reported semi-structured interviews and were not assessed using standardized or objective urological measures. Therefore, these observations should be interpreted with caution and cannot be considered definitive evidence of changes in autonomic or bladder function. Future studies should include validated bladder function scales and objective clinical assessments to more accurately evaluate potential autonomic effects of spinal neuromodulation interventions.

## 5. Conclusions

The application of a five-session intervention combining tSCS and RAGT is feasible and safe for the pediatric population with incomplete SCI. The most frequently observed adverse event was skin erythema at the site of current application. Preliminary findings suggest potential benefits in gait speed with this combined intervention after five training sessions with Lokomat and tSCS in children with SCI.

## Figures and Tables

**Table 1:** Demographic characteristics of the included participants.

	Age (Years)	Sex	Weight (kg)	Height (cm)	Motor Level of Injury	AIS Grade	Etiology	Time Since Injury (Months)	Assistive Device Use
Case 1	7	F	21	122	T11	C	Vascular	56	DAFO
Case 2	15	M	25	110	T3	C	Traumatic	18	Walker
Case 3	12	M	35	153	T3	D	Vascular	121	DAFO

*AIS—ASIA Impairment Scale, cm—centimeter, DAFO—dynamic ankle foot orthosis, F—female, kg—kilogram, M—male.*

**Table 2:** Distribution of adverse events.

Adverse Events	Distribution (Events/Percentage)	Severity Grading	Relationship with tSCS
Skin erythema	8 53%	75% mild 25% moderate	87.5% definite 12.5% probable
Skin itching or irritation	00%	-	-
Pain or burning sensation during stimulation	2 13%	100% mild	100% definite
Fatigue or drowsiness after the session	3 20%	100% mild	33.3% definite 33.3% possible 33.3% unrelated
Abdominal or trunk pain	1 6%	100% mild	100% possible
Vasovagal reaction	00%	-	-
Sweating, pallor, nausea, etc.	00%	-	-
Increased hypertonia	1 6%	100% mild	100% unrelated
Falls	1 6%	100% mild	100% unrelated
Bowel dysfunction	00%	-	-
Bladder dysfunction	2 13%	100% mild	50% definite 50% probable

**Table 3:** Functional outcomes.

Outcome		Time	#1	#2	#3
**MAS** **(RI/L)**	**Quadriceps**	Pre	3/3	3/3	2/2
		Post	3/3	3/3	2/2
	**Hamstrings**	Pre	3/3	3/3	2/2
		Post	3/3	3/3	2/2
	**Tibialis anterior**	Pre	2/2	2/2	2/2
		Post	2/2	2/2	2/2
	**Triceps surae**	Pre	1/1	1/1	1/1
		Post	1/1	1/1	1/1
**10MWT**		Pre	0.20 m/s	0.31 m/s	1.63 m/s
		Post	0.36 m/s	0.33 m/s	1.92 m/s
**LEMS** **(RI/L)**		Pre	8/8	11/13	21/17
		Post	8/8	11/14	20/17
**SATCo** **(S/A/R)**		Pre	7/7/5	7/7/5	7/7/7
		Post	7/7/5	7/7/5	7/7/7

*A: active, MAS: Modified Ashworth Scale; LEMS: lower-extremity motor score; L: left; R: reactive; RI: right; S: static; SATCo: Segmental Assessment of Trunk Control; 10MWT: 10-meter walk test.*

## Data Availability

The original contributions presented in this study are included in the article. Further inquiries can be directed to the corresponding author.

## Abbreviations

The following abbreviations are used in this manuscript:

10MWT	10-meter walk test
LEMS	Lower-extremity motor score
MCID	Minimal clinically important difference
MAS	Modified Ashworth Scale
RAGT	Robot-assisted gait training
SATCo	Segmental Assessment of Trunk Control
SCI	Spinal cord injury
tSCS	Transcutaneous spinal cord stimulation
